# Service implications of the revised 2022 National Institute for Health and Care Excellence (NICE) follow-up guidelines for stage IA–IIC melanoma

**DOI:** 10.1093/bjs/znad402

**Published:** 2024-01-25

**Authors:** Thomas D Dobbs, Mathew Jovic, Nattawan Ekakkaravichit, Stephen R Ali, John A G Gibson, Nader Ibrahim, Sarah Hemington-Gorse, Iain S Whitaker

**Affiliations:** Reconstructive Surgery and Regenerative Medicine Research Centre, Institute of Life Sciences, Swansea University Medical School, Swansea, UK; Welsh Centre for Burns and Plastic Surgery, Morriston Hospital, Swansea, UK; Reconstructive Surgery and Regenerative Medicine Research Centre, Institute of Life Sciences, Swansea University Medical School, Swansea, UK; St George’s University School of Medicine, St George's University, St George, Grenada; Reconstructive Surgery and Regenerative Medicine Research Centre, Institute of Life Sciences, Swansea University Medical School, Swansea, UK; Welsh Centre for Burns and Plastic Surgery, Morriston Hospital, Swansea, UK; Reconstructive Surgery and Regenerative Medicine Research Centre, Institute of Life Sciences, Swansea University Medical School, Swansea, UK; Welsh Centre for Burns and Plastic Surgery, Morriston Hospital, Swansea, UK; Reconstructive Surgery and Regenerative Medicine Research Centre, Institute of Life Sciences, Swansea University Medical School, Swansea, UK; Welsh Centre for Burns and Plastic Surgery, Morriston Hospital, Swansea, UK; Welsh Centre for Burns and Plastic Surgery, Morriston Hospital, Swansea, UK; Reconstructive Surgery and Regenerative Medicine Research Centre, Institute of Life Sciences, Swansea University Medical School, Swansea, UK; Welsh Centre for Burns and Plastic Surgery, Morriston Hospital, Swansea, UK

## Abstract

**Background:**

The 2022 National Institute for Health and Care Excellence melanoma guideline update made significant changes to follow-up. The aim of this study was to assess the impact these changes will have on a national melanoma cohort over a 5-year follow-up interval.

**Methods:**

Anonymized, individual-level, population-scale, linkable primary and secondary care National Health Service data for an 18-year interval (2000–2018) in Wales, UK were analysed. These data were used to predict the number of patients over a 10-year interval (2020–2030) that would be diagnosed with melanoma. Follow-up schedules for the 2015 and 2022 National Institute for Health and Care Excellence melanoma guidelines were then used to calculate the number of clinician-led appointments, the number of radiological investigations, and the total healthcare cost between 2025 and 2030, corresponding to a 5-year patient follow-up interval, for those with stage IA–IIC melanoma.

**Results:**

Between 2025 and 2030 it is predicted that implementation of the 2022 guidelines would lead to 21 122 (range 19 194–23 083) fewer clinician-led appointments for patients with stage IA–IIC melanoma. However, there would be a significant increase in the number of radiological investigations (7812; range 7444–8189). These changes would lead to a €2.74 million (€1.87 million–€3.61 million) reduction in the total cost of follow-up over the interval 2025–2030.

**Conclusion:**

Melanoma follow-up guideline changes will result in a substantial reduction in the number of clinical follow-up appointments, but a significant additional burden to radiological services. The overall cost of follow-up at a national level will be reduced.

## Teaser

Changes to the National Institute for Health and Care Excellence melanoma follow-up guidelines in 2022 will result in approximately 20 000 fewer clinician-led appointments over a 5-year interval in Wales. Despite the increase in radiology requirements, there will be a €2.74 million reduction in healthcare cost.

## Introduction

The worldwide incidence of primary cutaneous malignant melanoma has been rapidly increasing over the last 50 years^1^. Melanoma is now the fifth most common cancer in the UK, with over 16 000 new cases in 2018^2^. Significant advances in management, especially systemic therapy, have revolutionized outcomes^3^. The 5-year survival for early-stage disease is now close to 100%^4^, with 83% of patients with treated stage IV disease surviving 4 years, according to recent trial data^5^. Overall, 10-year survival across all stages of melanoma is now around 90%^6^.

As a result of both increasing incidence and survival, there are many people living with a melanoma diagnosis^4^. Many of these are in some form of clinical follow-up, with the primary aim being the early detection of recurrent disease or a new primary. Secondary aims include patient education, reassurance, and psychological support. However, this follow-up is burdensome for the health service and patients alike. There is also significant debate as to the best method and frequency of follow-up, with a variety of follow-up protocols used internationally^7^. This is largely the result of the complexity and risk of recurrence in different melanoma stages, as well as the way disease recurrence or a new primary can present. The annual risk of recurrence broadly rises with increasing AJCC stage^8^, with the risk of recurrence being low both in thin melanomas and after 10 years of follow-up^9^. In intermediate and thick melanomas, around 90% of recurrences occur in the first 3 years^10,11^. Furthermore, the risk of a new primary is also relatively low, at around 8% at 5 years, although this jumps significantly with other risk factors, such as increasing number of primary melanomas^12^. For some patients, intensive follow-up can lead to increased cancer-related anxiety. All of this, combined with financial constraints across healthcare systems, has led to extensive research into the best follow-up schedule to both promptly detect recurrent disease or a new disease, while, at the same time, not overburdening patients and the healthcare system.

Until recently, follow-up for melanoma in the UK was based on the 2015 National Institute for Health and Care Excellence (NICE) guidelines^13^. However, the international randomized controlled MELanoma FOllow-up (MELFO) study did demonstrate that a reduced frequency of follow-up for AJCC stage IB–IIC melanoma was both well tolerated by patients (no difference in anxiety, cancer worry, or mental health-related quality of life) and did not differ in progression-free or disease-specific survival^14–16^. Furthermore, the majority of recurrences were detected by patients and this was the same in both follow-up groups. This, in combination with studies supporting the use of imaging for follow-up^17^, led to the revision of the NICE guidelines in July 2022. These now involve reduced clinical follow-up, but an increase in imaging follow-up, for most melanoma disease stages. These changes could have profound implications for national skin cancer services, freeing up clinic capacity and potentially altering the cost of melanoma follow-up. Therefore, the aim of this study was to model the changes in the 2022 NICE guidelines on a national melanoma cohort.

## Methods

### Data source

Anonymized, individual-level, population-scale, linkable primary and secondary care National Health Service (NHS) data and national administrative data for an 18-year interval (2000–2018) in Wales, UK were analysed within the Secure Anonymized Information Linkage (SAIL) Databank^18–20^. This study was designed and reported in accordance with the REporting of studies Conducted using Observational Routinely-collected health Data (‘RECORD’) Statement^21^.

### Study population

All patients with a primary diagnosis of malignant melanoma between 2000 and 2018 were included. Cases were identified from the Welsh Cancer Intelligence and Surveillance Unit (WCISU) using ICD-10 codes (C43.0–C43.9) and ICDO-3 morphology codes (8720–8790)^22^ (where the O in ICDO stands for Oncology), validated in the authors’ previous work.

### Study design

After identification of cases, other data were extracted from linked data sets within the SAIL Databank, including stage at diagnosis, mortality data, and basic demographics. Stage at diagnosis was required to appropriately assign patients to the correct follow-up pathway, as per NICE guidelines. It was noted that in pre-2007 data, substages were less commonly documented (for example cases of either stage I or II disease were simply coded as either stage I or II, rather than the more extensive breakdown of IA/B etc.). This was significantly better in the post-2007 data, although some cases were still generically coded as stage I or II disease. To allow data to be analysed at a substage level, case adjustment was performed to reduce under-reporting of specific stage subgroups. The use of ratios allowed for these generically coded cases to be distributed into the appropriate specific substage. Linear regression of these data was then used to forecast the number of patients likely to be diagnosed with malignant melanoma in the following 10 years. As there is a known melanoma mortality rate (as well as a non-melanoma-specific mortality rate in the general population), not all patients diagnosed at any one time point will be alive for the duration of their 5-year follow-up or the 10-year forecasted interval in this study. Therefore, mortality data with the underlying cause of death from death certificate registrations were retrieved from the Annual District Death Extract (‘ADDE’) using ICD-10 codes. Kaplan–Meier plots were generated for all-cause mortality for all stages in the cohort over a 5-year interval. Using these, the probability of patients being alive at 1-year time points for a 5-year interval was calculated. From this, the number of patients alive for each year in the 10-year forecasted interval was adjusted to reduce over-reporting of the number of follow-up appointments needed.

This study compared the numbers of clinical follow-up appointments and radiological investigations between the 2015 and 2022 NICE melanoma guidelines^13,23^. *Table 1* shows the differences in the numbers of appointments and radiological investigations between these two sets of guidelines over a 5-year follow-up interval. To calculate the impact of these guideline changes, the number of new malignant melanoma diagnoses to 2030 (adjusted for death, as described above) was first forecasted using a linear regression model. From this, a 10-year interval (2020–2030) was selected from which the numbers of follow-up appointments and investigations for each substage for each year were calculated. The number of appointments each year was calculated individually for the 10-year interval. As the follow-up was started from zero follow-up in 2020, the first 5 years were for calculation purposes only. For each year, the number of patients who would be alive for the next 5 years was calculated and the remaining alive patients had the required NICE guideline follow up applied. This allowed the accurate calculation of the expected numbers of follow-up clinician-led appointments and radiological investigations from 2025 to 2030 for both the old and new NICE guidelines. A clinician-led appointment or radiological investigation was included in the analysis where it was given as an option in the relevant guidelines, even if ‘consider’ was suggested. Where radiological assessment was contingent on whether a patient had had a sentinel lymph node biopsy (SLNB) or not (that is ultrasonographic imaging of the draining lymph node basin in those considered for, but not having, an SLNB) a factor of the number of patients likely to be in this category was calculated from current national SLNB data. The current national SLNB data were applied to all patients in the cohort, as those early in the data set were treated before SLNB became routinely used. The number of people not having an SLNB was then used to calculate the number of people likely to undergo ultrasonographic imaging. In the new guidelines, a small number of patients are recommended to undergo MRI rather than CT imaging (those that are either less than 24 years old, pregnant, or have resected brain metastases). The number of patients this applied to in the study cohort was calculated, but, as the number corresponded to less than 3% of patients, separate MRI costings were not included, as this would have had a minimal impact on the overall cost. Finally, because of the small number of patients with stage III and IV disease at initial presentation, the more ‘personalized’ nature of the follow-up for these patients, and the relatively minor change in the 2022 follow-up guidelines for stage III disease, these patients were not included in the final analysis. The flow diagram in *Fig. S1* shows the study design.

**Table 1:** Comparison of the 2015 and 2022 NICE melanoma guidelines for patient follow-up

AJCC stage*	2015 NICE follow-up					Total imaging investigations	2022 NICE follow-up					Total imaging investigations	
	Year 1	Year 2	Year 3	Year 4	Year 5		Year 1	Year 2	Year 3	Year 4	Year 5	Ultrasonographic imaging of the draining lymph node basin	Whole-body CT imaging
	Clinician-led appointments per year						Clinician-led appointments per year						
IA	2	0	0	0	0	0	2	0	0	0	0	0†	0
IB	4	4	4	2	2	0	2	1	1	1	1	4†	0
IIA	4	4	4	2	2	0	2	2	1	1	1	5†	0
IIB	4	4	4	2	2	0	4	4	2	1	1	6†	8
IIC	4	4	4	2	2	0	4	4	2	1	1	6†	8

Values are *n*. *AJCC version 8 melanoma staging. †Option of ultrasonographic imaging for patients for whom a sentinel lymph node biopsy was considered, but not done.

### Cost analysis

The total cost difference between the 2015 and 2022 follow-up guidelines was calculated for the 2025–2030 modelled interval. Published NHS costs for clinician-led outpatient follow-up and radiological procedures were identified^24^. A time-series forecast using exponential smoothing allowed for a prediction of the cost of each procedure in the interval 2025–2030, accounting for previous cost increases (*Fig. S2*).

### English data analysis

While the focus of this analysis was Welsh patients, data were extrapolated to an English patient cohort for increased national generalizability. The number of patients diagnosed with melanoma in England were taken from the ‘Get Data Out’ resource (https://www.cancerdata.nhs.uk/getdataout/skin). The number of patients with melanoma in England was forecasted to 2030 in the manner described above. The same percentage of patients in Wales with stage IA–IIC melanoma relative to the total number was then applied to the English cohort. The same analysis described above was then performed to calculate the total number of appointments and cost difference between both iterations of the NICE guidelines.

### Statistical analysis

Data were extracted in the SQL query platform IBM DB2 (IBM, Armonk, NY, USA). A combination of analysis software was used. Data manipulation and survival analysis were undertaken in RStudio (Posit, Boston, MA, USA), linear regression models were constructed in IBM SPSS^®^ (IBM, Armonk, NY, USA), analysis of follow-up appointments was carried out using Microsoft Excel (Microsoft Corp., Redmond, WA, USA), and figures and cost forecasting were computed in Wolfram Mathematica (Wolfram Research, Oxford, UK). Statistical significance was assumed with a *P* < 0.050 and an R-squared value of greater than or equal to 0.7 was used to indicate a good fit for linear regression models.

### Ethical approval

This study was approved by the SAIL independent Information Governance Review Panel (IGRP) (project 0593). In accordance with Health Research Authority guidance, NHS research ethics committee approval is not mandatory for studies using only anonymized data.

## Results

A total of 11 935 patients were identified as having had a diagnosis of malignant melanoma in Wales between 2000 and 2018, of which 9715 cases were analysable. Overall, 50.6% of patients were male, with a mean age at diagnosis of 67 years. A more detailed breakdown of demographics is presented in *Table 2*. In *Fig. S3*, the forecasted number of new diagnoses between 2020 and 2030 is shown. Kaplan–Meier curves for survival in this 5-year interval, based on data from 2000 to 2018, are shown in *Fig. S4*. These data allowed an accurate calculation of the number of patients alive at any point during follow-up and therefore reduced over-reporting.

**Table 2:** Demographic data of patients diagnosed with malignant melanoma between 2000 and 2018 in Wales, UK

	Stage 1	Stage 2	Stage 3	Stage 4	Percentage
**Age (years)**					
<25	96	25	9	0	1.3
25–49	1609	303	86	57	21.2
50–75	3285	1198	304	190	51.2
>75	1276	976	191	109	26.3
Percentage	64.5	25.8	6.1	3.6	
Median (interquartile range)	61 (48–72)	71 (60–80)	69 (56–77)	68 (55–77)	
**Sex**					
Male	2950	1412	332	220	50.6
Female	3313	1090	258	140	49.4
**Anatomical location**					
Head and neck	1038	599	111	50	19.0
Trunk	2164	718	186	87	33.3
Upper limb	1475	569	110	36	23.2
Lower limb	1528	589	162	40	24.5

Values are *n* unless otherwise indicated.

### Difference in the number of appointments between new and old guidelines

**Fig. 1:**
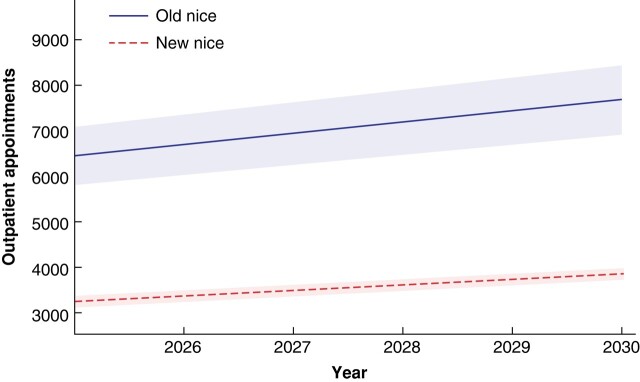
Modelled number of outpatient appointments per year in the follow-up interval of 2025–2030 for the 2015 and 2022 NICE melanoma guidelines

*Figure 1* shows the difference in the number of appointments for the interval of 2025–2030 between the old NICE melanoma guidelines and the newly implemented guidelines. A total of 42 358 (range 41 140–43 634) appointments would be needed using the old guidelines and 21 236 (range 20 551–21 946) with the new guidelines, a reduction of 21 122 (range 19 194–23 083) appointments.

### Number of radiological investigations

The 2015 guidelines did not specify radiological investigations for patients with stage IA–IIC melanoma, so it was not possible to calculate an accurate figure for the number of such investigations performed under these guidelines. Thus, the only patients undergoing imaging in this cohort would have been those for whom there was clinical concern. For the purposes of this study, the number of radiological investigations under the 2015 guidelines for patients with stage IA–IIC melanoma was assumed to be zero.

Under the 2022 guidelines, ultrasonographic imaging is indicated for patients with stage IB–IIC melanoma where an SLNB was considered, but not done. National SLNB data demonstrated that 83.2% of the eligible cohort were undergoing SLNB, suggesting 16.8% of patients would have routine ultrasonographic imaging, as per new NICE guidelines. This is comparable to published data^25,26^. Upon calculation, it was found that the total number of radiological investigations according to the 2022 guidelines in a forecasted 5-year interval (2025–2030) would be 7812 (range 7444–8189), split into 5512 (range 5204–5827) CT imaging investigations and 2300 (range 2240–2362) ultrasonographic imaging investigations (*Table 3*).

**Table 3:** Modelled number of radiological imaging investigations in the follow-up interval of 2025–2030 for the 2022 NICE melanoma guidelines

Year	Total CT imaging investigations (range)	Total ultrasonographic imaging investigations (range)
2025	838 (791–885)	350 (341–360)
2026	872 (821–922)	364 (354–374)
2027	902 (854–953)	377 (367–387)
2028	934 (881–988)	389 (379–400)
2029	965 (912–1022)	403 (392–414)
2030	1001 (945–1057)	417 (406–428)
Total (2025–2030)	5512 (5204–5827)	2300 (2240–2362)

Values are *n*.

### Cost difference

**Fig. 2:**
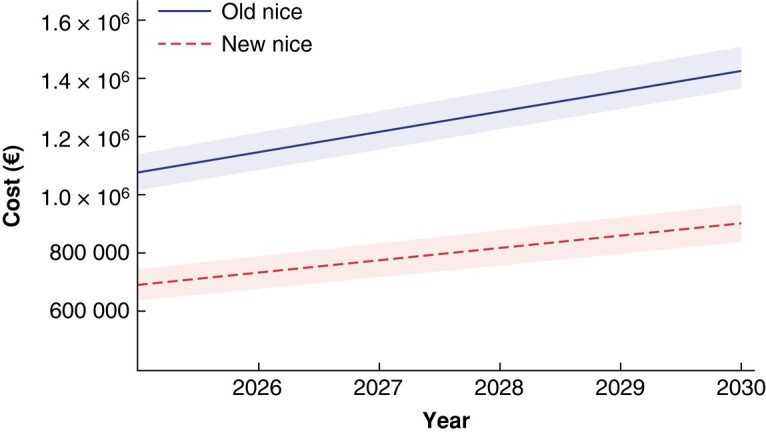
Total cost of melanoma follow-up for the interval 2025–2030, based on the 2015 and 2022 NICE melanoma guidelines

The total cost of outpatient clinical follow-up for the years 2025–2030 under the new guidelines would be €3.77 million (range €3.53 million–€4.01 million), an almost 50% reduction compared with the costs under the 2015 melanoma guidelines for the same patient cohort. However, there would be a substantial increase in the cost of radiological follow-up. Under the new guidelines this would equate to €1 million (range €8469711–€1.18 million) for the years 2025–2030. Therefore, the combined cost of all follow-up (both outpatient clinics and radiology) under the 2022 guidelines would be €4.77 million (range €4.37 million–€5.20 million), compared with €7.50 million (range €7.06 million–€7.98 million) using the 2015 guidelines, representing a cost difference of €2.74 million (range €1.87 million–€3.61 million) (*Fig. 2*).

### Estimated English results

In England, there would be a predicted reduction of 165 977 appointments (range 161 788–170 424) over the 5-year study interval. There would be a predicted total reduction in the cost of melanoma follow-up of €29 418 469 (range €23 956 614–€34 974 607) over a 5-year interval.

## Discussion

This is the first study to assess the national impact of the introduction of new melanoma NICE follow-up guidelines on patients with stage IA–IIC disease. This work has shown that, while there will be a substantial reduction in the number of outpatient follow-up appointments required, there will be a significant increase in the requirement for radiological investigations of these patients. In a modelled 5-year interval between 2025 and 2030 there will be an approximately 50% reduction in clinician-led follow-up, but the addition of 7812 imaging investigations, with the introduction of the 2022 guidelines.

These changes will have a number of important implications. The cost of imaging will be significantly increased under the 2022 guidelines, but the overall cost of melanoma follow-up will be significantly decreased. Over a 5-year interval, the new guidelines will save a predicted €2.75 million in Wales, a cost reduction of 36% compared with the old guidelines. This figure would be approximately €29 million in England. This is important, considering the current financial pressures affecting healthcare systems. Furthermore, the 50% reduction in clinician-led follow-up (21 122 fewer appointments over a 5-year interval) will release both significant time and resources. Current waiting times for a new diagnosis of cancer make it difficult to meet national targets^27^, with waiting lists still impacted by the COVID-19 pandemic^28^ and due to a shortage of healthcare professionals^29^. The release of appointments should permit new patient referrals to be seen on time, improving patient care and long-term outcomes. While cost and clinician-led clinical follow-up reductions will be seen, the large increase in routine imaging follow-up needs to be considered. This will have a significant impact on radiology services across the country, which are already struggling with their workload. Local skin cancer services and multidisciplinary teams will need to work with colleagues in radiology, by looking at workforce planning and the potential integration of artificial intelligence, to best manage this additional burden^30^.

Follow-up is an important component of cancer management. Not only can it help detect a new primary or recurrent disease, but secondary benefits, such as patient education and psychological support, are also important. However, there is a realization that current follow-up methods may not be satisfactory in addressing these needs. The new NICE guidelines and previous studies are beginning to open the discussion as to how far the reduction of in-person follow-up can go. Evidence from the MELFO study suggests that there was no difference in the recurrence rate between follow-up schedules and that approximately 70% of recurrence was detected by the patients^15^. This would suggest that, for many patients, routine melanoma follow-up is not fulfilling one of its main aims, that is the detection of recurrence by clinicians at follow-up. Metastatic recurrence (especially at an early stage) is also difficult to detect during a clinical examination. This is where an increase in imaging will be beneficial. An increase in the use of novel technologies should allow the safe reduction of in-person follow-up, while having added patient benefits. Remote patient monitoring of symptoms with routine electronic patient-reported outcome measures may allow the early identification of patients who need a clinical review, while limiting the number of routine appointments^31^. This could also be provided as part of a wider package of digital health and well-being support, from which patients could access lifestyle advice and mental health support, which is currently under-resourced in most healthcare settings. Cost savings, resulting from reduced clinical follow-up, could be further invested in improved patient care. This aligns with the delivery model of value-based healthcare, which forms a key backbone of healthcare delivery^32^.

As with any study based on routinely collected health data, there are risks of missing or mis-coded data. This has been mitigated as much as possible by identifying patients in data sets managed by the WCISU, a national cancer registry to which there is mandatory reporting of cases. In circumstances for which the 2022 guidelines used terms such as ‘consider’ in relation to imaging, it was opted to include these in the procedural burden and, as a result, the number of imaging follow-ups may be overestimated. In practice, while these investigations may not be done for everyone who could be eligible, for completeness and the truest picture of the potential overall cost difference, it was assumed that all patients will have all recommended imaging investigations. Furthermore, the number of appointments is modelled on the guidelines and cannot fully represent the number of appointments that actually take place, that is personalized and more intense follow-up that is typically employed in those with stage III and IV disease undergoing systemic therapy. It is for this reason that stage III and IV patients were not included in the analysis, although this unlikely significantly impacted the total number of appointments or the associated cost. To strengthen the study, adjustments were made for loss to follow-up, based on death of patients during the 5-year follow-up interval. The loss to follow-up may be a slight overestimation, as the all-cause mortality rates are based on retrospective data and therefore may not fully reflect the current picture in the era of effective adjuvant treatment. It was also not feasible to calculate the number of patients who cross between stage I or II disease into stage III or IV disease, during their follow-up interval, and therefore there might be a slight underestimation of the number of appointments these patients would have over a lifetime. Finally, caution must be applied to the English data analysis, which is only included for illustrative purposes. The authors would recommend a formal analysis of English patient numbers in the same manner as the Welsh patient analysis.

The melanoma follow-up guideline changes by NICE in 2022 will result in substantial reductions in both the number of clinician-led appointments and the overall associated cost of melanoma follow-up for those with stage IA–IIC disease. Follow-up that is less intense, with remote patient monitoring, is likely to have significant patient and healthcare-system benefits. It will be important that the increased appointment availability and available money are used wisely and that patients feel safe and reassured regarding the new method of follow-up.

## Supplementary Material

znad402_Supplementary_Data

## Data Availability

All data are held within the SAIL Databank and will be made available on request, provided that appropriate approvals regarding data security are in place. A pre-analysis plan was not formally published.
