# Supplementary material for: Service implications of the revised 2022 National Institute for Health and Care Excellence (NICE) follow-up guidelines for stage IA–IIC melanoma
Source: Br J Surg. 2024 Jan 25;111(1):znad402. doi: 10.1093/bjs/znad402 (PMC10810063; doi:10.1093/bjs/znad402)

**Title: National service implications of the revised 2022 NICE follow-up guidelines in stage 1A – 2C melanoma**

Authors : Thomas D Dobbs^1, 2^, Mathew Jovic^1^, Nattawan Ekakkaravichit^3^, Stephen R Ali^1, 2^, John AG Gibson^1, 2^, Nader Ibrahim^1, 2^, Sarah Hemington-Gorse^2^, Iain S Whitaker^1, 2^

1. Reconstructive Surgery and Regenerative Medicine Research Centre. Institute of Life Sciences, Swansea University Medical School, Swansea, UK
2. Welsh Centre for Burns and Plastic Surgery, Morriston Hospital, Swansea, UK
3. St George’s University School of Medicine, St George, Grenada

**Corresponding author.**

Mr Thomas Dobbs BM BCh, MA(Oxon), PhD, FRCS(Plast)

Reconstructive Surgery & Regenerative Medicine Research Centre, Institute of Life Sciences, Swansea University Medical School, Swansea SA2 8PP, United Kingdom

Tel: 01792205678

Email: tomdobbs@doctors.org.uk

**Supplementary Materials - Index**

**Supplementary Figure 1:** Study flow diagram demonstrating the steps of data analysis.

ICD – International Classification of Diseases. ICDO – International Classification of Diseases for Oncology. SLNB – sentinel lymph node biopsy. Stage 3 and 4 patients excluded from final analysis.

Page 7


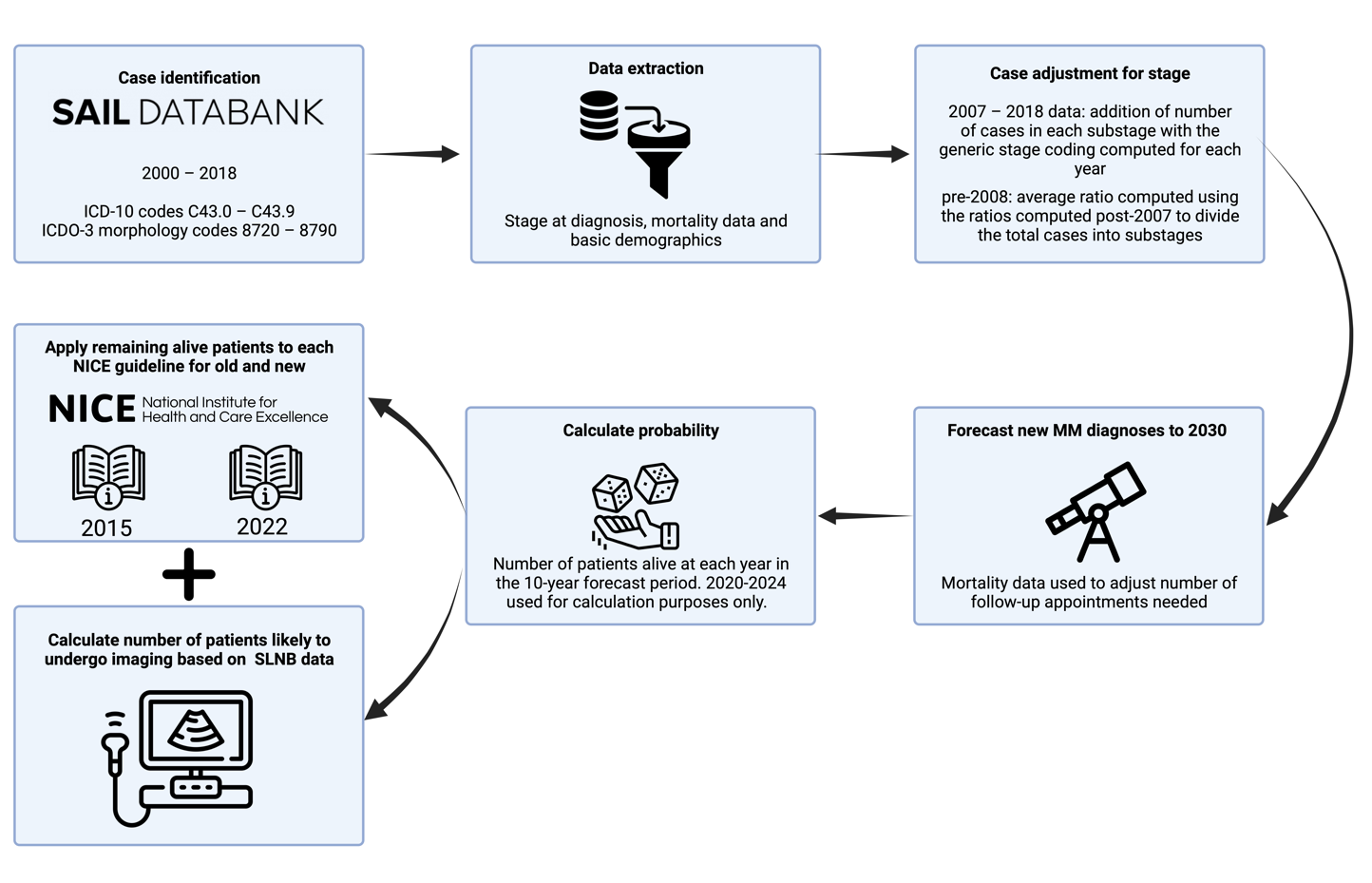


**Supplementary Figure 2:** Forecasted NHS costs per radiological imaging and clinician led (CL) appointment.

Page 7


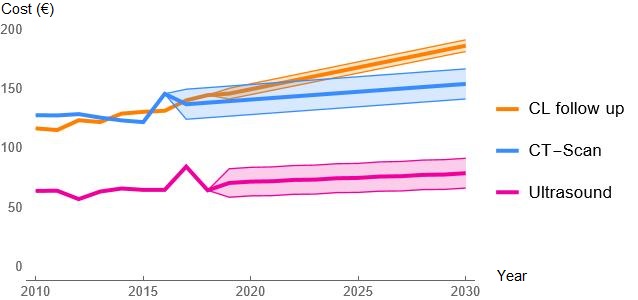


**Supplementary Figure 3:** Forecasted number of people living with malignant melanoma in Wales each year between 2020 – 2030.

Page 8


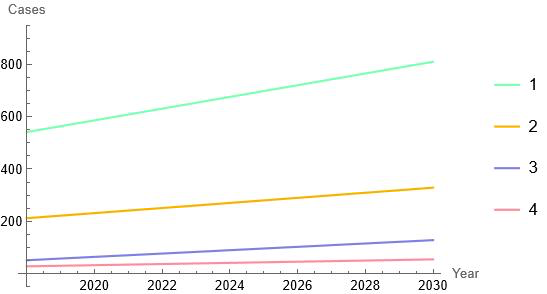


**Supplementary Figure 4:** Kaplan Meier curves indicating the probability of survival for a 5-year post diagnosis period per stage.

Page 8


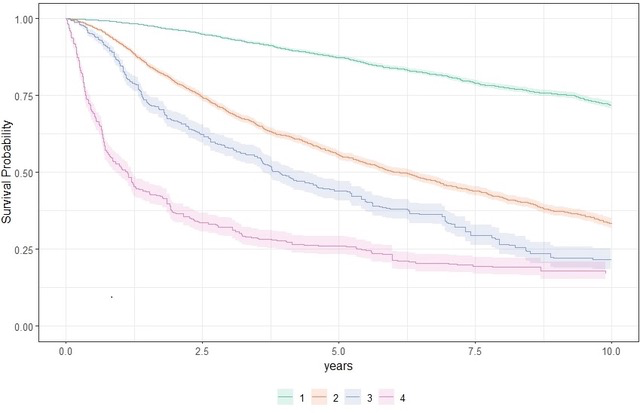

Supplement: znad402_Supplementary_Data [file znad402_supplementary_data.zip › Supplementary-materials.docx]
