# Supplementary figures and images for: Service implications of the revised 2022 National Institute for Health and Care Excellence (NICE) follow-up guidelines for stage IA–IIC melanoma
Source: Br J Surg. 2024 Jan 25;111(1):znad402. doi: 10.1093/bjs/znad402 (PMC10810063; doi:10.1093/bjs/znad402)

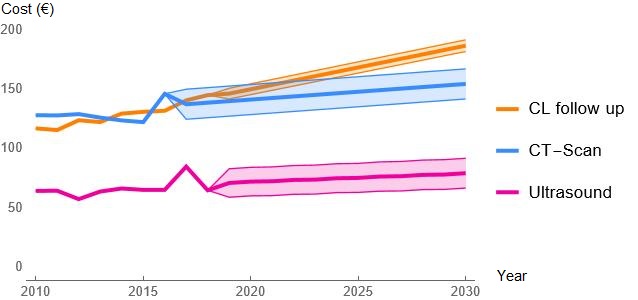

Supplement: znad402_Supplementary_Data [file znad402_supplementary_data.zip › Supp Fig 2_euro.jpeg]
